# Marchiafava Bignami Disease: A Rare Neurological Complication of Long-Term Alcohol Abuse

**DOI:** 10.7759/cureus.30863

**Published:** 2022-10-30

**Authors:** Shailja Singh, Vasant Wagh

**Affiliations:** 1 Department of Medicine, Datta Meghe Institute of Medical Sciences, Wardha, IND; 2 Department of Community Medicine, Datta Meghe Institute of Medical Sciences, Wardha, IND

**Keywords:** treatment, diagnosis, marchiafava–bignami disease, demyelination, neurological disorder, alcoholism

## Abstract

Marchiafava Bignami disease is a demyelinating and necrotic disease of the central nervous system in chronic alcohol users and malnourished patients. The primary characteristic lesion of this disease is present in the corpus callosum in the form of its necrosis, but plenty of evidence suggests that it can also affect other parts of the brain. The main pathophysiology revolves around the consumption of alcohol and its ability to cause thiamine depletion in the body and hinder various metabolic pathways. There is also a hindrance in myelin synthesis, which further damages the signal transmission leading to an array of symptoms and signs. It is present in different degrees in patients in the form of different stages, namely acute, subacute, and chronic. The diagnosis of the disease becomes tough as the presenting symptoms are very generic and vague. Before the innovation of advanced imaging techniques, it was mainly a finding during an autopsy, but presently it can be diagnosed by a well-taken history and imaging techniques which can help to rule out other diseases having a similar clinical presentation. The gold standard for the diagnosis of the disease is using magnetic resonance imaging (MRI) techniques to visualize the lesions present in the corpus callosum and other areas, but other methods like computed tomography (CT) are also used. The prognosis of the disease is influenced by many factors, and it varies greatly. Some factors such as broad involvement of the cerebral cortex and severe disturbances in consciousness are indicative of a poor prognosis. The differential diagnosis consists of other alcohol use disorders like Wernicke’s encephalopathy, neoplastic conditions, and multiple sclerosis, to mention a few. Each one should be carefully eliminated before finalizing the diagnosis. The treatment of the disease is not concrete, but evidence shows improvement with specific interventions.

## Introduction and background

A highly rare toxic demyelinating and necrotic condition of the central nervous system (CNS) known as Marchiafava Bignami disease (MBD) is characterized by corpus callosum degeneration [1]. Two Italian pathologists initially explained it in 1903, namely Ettore Machiafava and Amico Bignami [2]. The initial description provided by these pathologists was believed to be connected to the frequent intake of inexpensive chianti, a type of wine. They gave examples of persons with alcohol use disorders who passed away from convulsions and comas and had corpus callosum necrosis upon autopsy. The vast majority of the afflicted have a long-term history of chronic alcohol use and malnutrition, are male and are between the ages of 40 and 60 years [3,4]. It has also been seen in patients who are non-alcoholic and also are not suffering from malnutrition [5,6]. Even in the absence of traditional clinical symptoms, early diagnosis of lesions in MBD is now possible because of advancements in contemporary brain imaging techniques. Because of this, medical professionals can now consider the presence of this disease even in the emergency room and start thinking about effective treatment options [7]. The corpus callosum is symmetrically involved in the standard MRI, with the body of the corpus typically affected before the renu and splenium [8]. There is growing evidence that MBD injuries can affect other parts of the brain along with the corpus callosum, including the basal ganglia, subcortical areas, cerebral lobes, and white matter in the hemisphere. MBD develops as significant neurological dysfunction and has a dismal prognosis when other brain regions are affected [9].

## Review

Aetiopathogenesis 

Although the precise pathophysiology is as yet unknown, it is typically blamed on a vitamin B complex inadequacy [10]. MBD has been well-identified in individuals who were either malnourished or alcoholic. Its association with nutritional deficits in alcoholics is especially notable because severe alcohol consumption is often recognized to promote thiamine wastage even in the absence of caloric starvation [7]. Breach of the blood-brain barrier, necrosis, cytotoxic edema, and demyelination are a few potential causes associated with MBD [1]. When hyperintense lesions are observed on diffusion-weighted imaging (DWI) in the early stages, cytotoxic edema is suggested as a potential underlying mechanism, while necrosis and demyelination may be involved in the later stages [11]. Other possible etiology includes callosal myelinolysis as a consequence of ketoacidosis brought on by alcoholism or diabetes mellitus [12] and non-alcoholic malnourishment as a result of gastric bypass surgery [13]. Various non-alcoholic illnesses like sepsis, sickle cell disease, carbon monoxide poisoning, cerebral malaria, and cardiac cancer surgery have all been linked to it [14,15].

Pathophysiology Related to Alcohol Abuse

Alcohol use is a significant contributor to CNS diseases. Alcohol affects the expression of proteins that attach cytoskeletal elements in the white matter, inhibits neuronal plasticity, changes neurotransmitter function, and causes disruptions to lipid metabolism [16]. Aldehyde dehydrogenase (ADH) is a mechanism that can metabolize ethanol in the CNS in an oxidative manner, which results in oxidative stress disease [17]. Lack of catechol-O-methyl transferase activity leads to increased activity of catecholamine neurotransmitters like dopamine, which then causes delusions, hallucinations, and delirium in people deficient in the B1 vitamin and this affects the carbohydrate metabolism process and lowers the amount of adenosine triphosphate that is available [18]. Additionally, there is also a decrease in the production of other neurotransmitters like GABA, acetylcholine, and glutamate, all of which might be connected to insufficient pyruvate dehydrogenase activity. This failure in glutathione and myelin synthesis impairs the capacity of neurons to transmit signals and defend themselves against oxidative stress [19]. High myelin concentration in the main white matter commissure which links both hemispheres and supports the transmission of motor, sensory, and cognitive information can be used to explain the damage to the corpus callosum [20].

Histopathology

Almost only during a post-mortem autopsy is a histologic diagnosis carried out. Ironside et al. conducted a review of 89 autopsy cases reported from 1898 to 1959 in 1961 and concluded that there is an individual and racial susceptibility to this disease. Since then, a rising number of non-fatal cases have been documented, and more entities that closely resemble the condition have also been identified [7]. Necrotizing or cystic lesions can be macroscopic histopathologic characteristics in the corpus callosum, particularly in the genu and body. White matter necrosis, gliosis, numerous macrophages (with limited inflammatory reaction), small perivascular lymphocytes (mainly CD3+ T cells), foamy histiocyte invasion (marked by CD68 and CD163), and eminent demyelination (with comparative sparing of the axons) can all be seen under a microscope. Histopathological studies by Lechevalier et al. also described interhemispheric disconnections and that this demyelination can also extend symmetrically to the oligodendrocytes [21,22].

Clinical features

This disease has a wide clinical spectrum, which makes diagnosis challenging, and its presentation is non-specific [10]. Patients with specific typical neurological symptoms and a history of prolonged alcohol abuse, malnutrition, or both should be evaluated for MBD. It might have an acute, subacute, or chronic manifestation. Patients experience a variety of neurological symptoms during the acute phase, including confusion, dysarthria, limb hypertonicity, ataxia, and delirium/coma [1,10]. Confusion, dysarthria, aberrant behavior, somnolence, and visual disruption are also symptoms of the subacute type [1]. Interhemispheric disconnection syndrome, increasing dementia, and aberrant behavior are all part of the chronic presentation. Hemialexia, limb apraxia, unilateral agraphia, and tactile agraphia is also seen. It's important to distinguish between Alzheimer's disease and the chronic phase of MBD, which manifests as dementia [23]. It is significant to note that cognitive abnormalities may be related to microhemorrhages. The sites of microbleeds and the effects on the various cognitive domains appear to be related anatomically, and microbleeds can degrade cognition. Executive dysfunction, memory (both short-term and long-term), language, and spatial awareness are all predicted by microbleeds [24]. Some patients have also seen an intermediate form that starts acutely before regressing to a chronic state [25]. Mild clinical indicators like hemiparesis, headache, depression, dizziness, psychotic and emotional symptoms, and apathy are recognized as first manifestations because MBD's clinical manifestations are not unique to this illness. However, MBD may develop into a coma or even death if diagnosis and treatment are delayed [1]. Gaze palsy or diplopia, incontinence, primitive reflexes, dysarthria, rigidity, evidence of disconnection or split-brain syndrome, mutism, and sensory symptoms were all shown to be associated with MBD in research by Hillbom et al. that examined 122 reports with data on 153 people who were established cases MBD [7]. Two distinct clinicoradiological categories were suggested by Heinrich's recent classification of MBD. Type A is characterized by immediate to subacute development of consciousness disturbance, seizures, pyramidal tract symptoms, hypertonia of limb, and extremely severe swelling of the corpus callosum on T2 weighted MR sequences; poor prognosis [10,26]. Type B includes hyperintense lesions on T2-weighted MR sequences in part affecting the corpus callosum, dysarthria, gait disturbance, evidence of disconnection between hemispheres, and normal or mildly compromised level of consciousness. The prognosis for type B is good, and reversible lesions point to underlying edema rather than demyelination [2]. According to post-mortem studies, morel's laminar sclerosis, Wernicke's encephalopathy, and central pontine myelinolysis have all been linked to MBD as other chronic alcohol misuse symptoms [27,28]. Even though the callosal lesions have been identified as the condition’s defining feature, only a limited number of patients with MBD also show anomalies in signal intensity in the cerebral parenchyma. The temporal and lateral frontal lobes are most frequently damaged.

Diagnosis 

**Figure 1:**
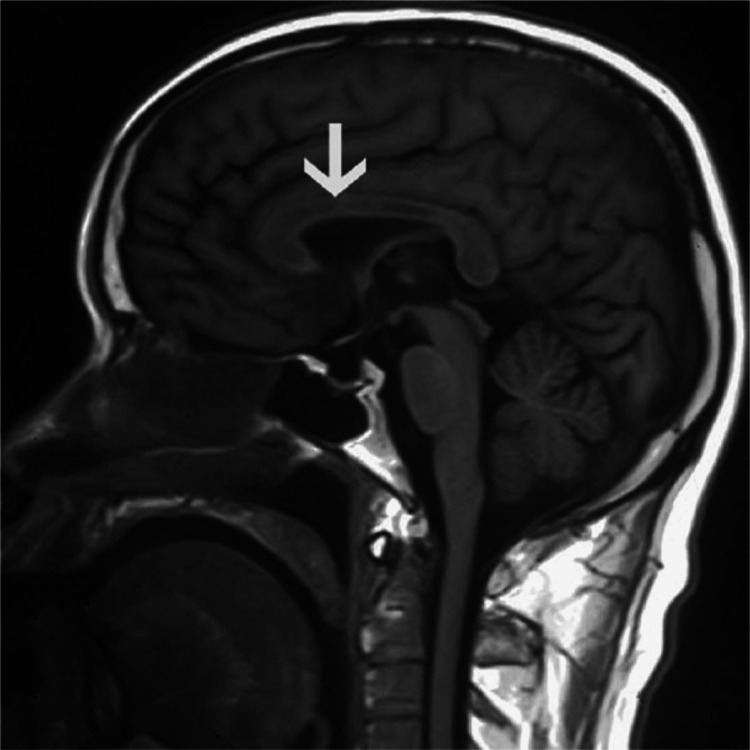
MRI T1-weighted sagittal view showing the sandwich sign - hypointense center of corpus callosum but normal area around it. Reference [30] is licensed under CC BY-NC 4.0

**Figure 2:**
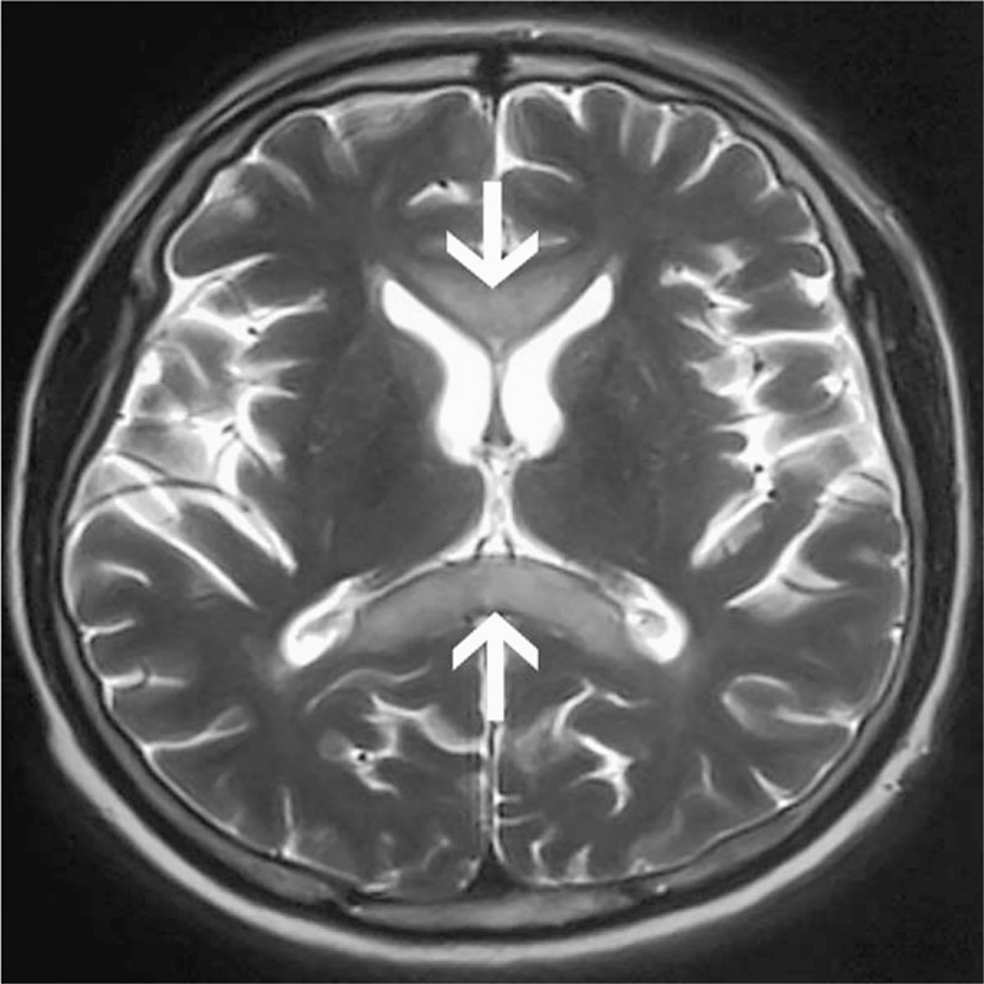
MRI T2 weighted Reference [30] is licensed under CC BY-NC 4.0

**Figure 3:**
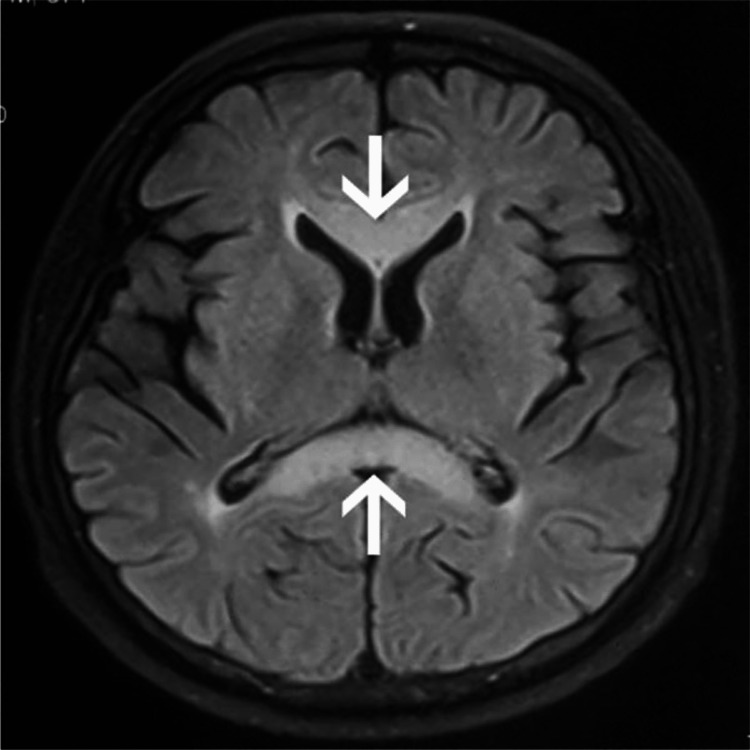
FLAIR showing abnormal hypointensity in corpus callosum Reference [30] is licensed under CC BY-NC 4.0

Before the advancements in imaging techniques, the diagnosis of MBD was almost exclusively on autopsy. Presently evaluation banks extensively on imaging results and correlation with an in-depth and complete history and physical exam, MRI is the gold standard of imaging techniques. The subject's disease stage may have an impact on the precision of the diagnosis and the results and effectiveness of the treatment. The “sandwich sign,” a symmetrical lesion encompassing the body of the corpus callosum's central region but leaving out the ventral and dorsal layer, is the hallmark of acute MBD [10], where chronic lesions may develop into well-defined “cavitation,” except in cases of subacute hemorrhage, in which the lesions can appear as isodense or hyperdense. The CT scan reveals that the lesions are hypodense [3]. The impaired area has edematous changes which can be present along with or without demyelination [1], and the lesions are hypointense on T1-weighted image (T1WI), hyperintense on T2-weighted image (T2WI) and fluid-attenuated inversion recovery (FLAIR), showing diffusion restriction on DWI [10,29]. The corpus callosum and bilateral periventricular white matter are diffusely hyperintense on T2-weighted imaging, and there is a correspondingly hyperintense T1 signal. In DWI, which is an MRI technique with high sensitivity for showing cytotoxic edema and infarcts, there is evidence of reduced diffusion in the middle layer of the corpus callosum's splenium and bilateral peritrigonal white matter, as well as a corresponding drop in signal [27]. FLAIR scans of the corpus callosum revealed its central hypointensity and surrounding hyperintense rim. Furthermore, variations in cortical-subcortical signal intensity were also seen, with the right frontal lobe showing hypointensity on T1WI and hyperintensity on T2WI [8]. Various imaging techniques include MR spectroscopy (MRS), which exhibits a higher level of choline and an increased ratio of choline to creatine (Cho/Cr) during the acute period. The acute or subacute stage of demyelination is often when a lactate peak is observed. Single-photon emission computerized tomography (SPECT) scans reveal bilateral cerebral blood flow decrease [2]. Patients with MBD have areas of low T1 signal intensity, high T2 and FLAIR signal intensity, and either or both related lesions in the cerebral white matter on MRI [8]. Several investigations have shown brain hypometabolism and hypoperfusion using (18F)-2-fluoro-2-deoxy-D-glucose PET, technetium 99m hexylmethylpropylene aminoexime SPECT, and/or N-isopropyl-p-(123I) iodoamphetamine SPECT. These findings are exclusively present in MBD patients who have had a poor or only partial recovery and represent a late disease state with persistent brain damage [7]. To sum up, from a radiological perspective, it is significant to note that MBD has a specific MRI appearance. Symmetric T1WI hypointensities and symmetric T2WI hyperintensities throughout the whole corpus callosum (CC) show a total loss of myelin with the replacement of the region by the cyst. Lesions in the internal capsule, cortex and middle cerebellar peduncles also occur less commonly. After the post-acute period, contrast enhancement of CC resolves. CC symmetrical atrophy with focal hypointensities on T1 and correlating focal hyperintensities on T2 (which appears to suggest progressive demyelination, regional necrosis, along with the formation of cyst -damage to myelin at the rim with a central necrotic area) cortical and subcortical atrophy, cerebellar vermix, and hemispheric atrophy, and cerebellar vermix atrophy seen [24]. Laboratory exams can also be employed to aid the diagnosis further; like serum electrolytes to rule out electrolyte conditions that can result in coma, impaired consciousness, and convulsions, to evaluate liver damage serum transaminases and bilirubin testing can be done, to rule out hypo/hyperglycemia serum glucose levels can be checked. A complete blood count can also be carried out to identify infectious/inflammatory reasons, determine hemoglobin and platelet levels, and detect alcohol addiction disorder if there is any evidence of macrocytosis or macrocytic anemia. To rule out abuse of other substances toxicology screening should be carried out and to determine if there is a systemic or CNS infection serum and spinal fluid infectious serology panel can be done (Figures 1-3) [20].

Differential Diagnosis 

Other chronic, alcohol-related disorders are part of the MBD differential diagnosis. Wernicke's encephalopathy, which is also an alcohol-related condition that can co-occur with MBD and manifest as ophthalmoplegia, nystagmus, ataxia, and disorientation, must be separated from MBD [1]. Infarction of the recurrent artery of Heubner, neoplastic conditions like lymphoma or astrocytoma, demyelinating conditions like multiple sclerosis (MS), progressive multifocal leukoencephalopathy, or acute disseminated encephalomyelitis are among the other differential diagnoses. MS must be ruled out because it is, without a doubt, the most frequent. Upon brain CT scans or MRI, MBD displays symmetric and limited edematous areas similar to MS [2]. MBD patients differ from individuals with an MBD mimic condition in several ways. Only individuals with malnutrition or alcoholism have been diagnosed with MBD, and this condition's link to nutritional deficiencies in alcoholics is especially notable because excessive alcohol consumption is often recognized to promote thiamine loss even in the absence of caloric malnutrition. In contrast, hypoglycemia, cerebral infection, high altitude sickness, antiepileptic drug withdrawal, epilepsy, and systemic lupus erythematosus are more frequently linked to MBD mimics than these other conditions. Second, the range of symptoms and manifestations varies. In contrast to the indications and symptoms of illnesses that mimic MBD, which are typically moderate and resolved within a week, subjects with MBD frequently have severe signs and symptoms as well as a sluggish rate of recovery. Both MBD and the MBD imitators exhibit delirious behavior, disordered consciousness, and seizures; however, the MBD mimics appear to experience seizures even more frequently. While single splenial lesions are only present in one-third of MBD cases, the splenium typically houses the lesions in MBD mimics [7].

Treatment 

There is no particular treatment for MBD. Randomized trials for the treatment of MBD are impractical because the condition is not common. Reviewing previously published case reports is the only practical study design that can give light on the effectiveness of treatment. Clinical recovery is accelerated by early diagnosis, thiamine, vitamin B complex, and folic acid treatment [10]. A study by Dong et al. claims that patients with substantial neurocognitive abnormalities and disturbances of consciousness appear to have a bad outcome, and lesions may not go away. The findings of this study show that individuals with severe alterations of consciousness as measured by GCS, extra callosal lesions, cerebral lobe damage, and heavy alcohol intake do not have a favorable outcome [31-34]. According to a study by Hillbom et al., within two weeks of the onset of symptoms, those who received thiamine treatment had a considerably reduced rate of poor outcome compared to those who received treatment later [7].whereas administration of steroids had no discernible effect on outcomes. This study makes two suggestions for the therapy of MBD based on the available information. First, DWI is the preferred technique for imaging an individual with MBD, diffusion tensor imaging (DTI), a relatively new type of MRI, and fiber tracking which uses DTI to make a 3D reconstruction of the brain to assess the neural tracts are the best ways to track recovery. Second, immediate intervention with parenteral thiamine appears to be necessary due to the high prevalence of thiamine deficit among chronic alcohol users and undernourished individuals [7]. But generally, corticosteroids are also administrated as they are postulated to reduce inflammatory edema, stabilize the blood-brain barrier, and decrease the production of leucocytes. However, evidence suggests that patients can achieve full recovery with adequate thiamine therapy [35]. The precise method through which thiamine therapy for MBD might work is unknown. Thiamine deficiency makes it harder for cells to control osmotic gradients, which could lead to cytotoxic edema. According to a theory, oligodendrocytes, which are normally found in white matter, may be more susceptible to osmotic stress when they are close to grey matter. [36]. Thiamine can be given as treatment in normal saline or 5% dextrose [37] also B Complex pills if needed. Amantadine is another drug that is used, the mechanism is unknown, and no rigorous data are backing up the use, but it may prove helpful in relieving the patient’s symptoms due to its dopaminergic effect. Folic acid can be given orally for the treatment or prevention of megaloblastic and macrocytic anemias [20]. In a case report by Staszewski et al., the use of amantadine with folic acid was also mentioned. The patient recovered after receiving amantadine together with thiamine, vitamin B-12, and folate. In a different instance, as described by Kikkawa et al., it was claimed that the delivery of high-dose corticosteroids came first [27]. However, the outcomes of MBD may vary drastically, even with a set treatment plan. 

Prognosis

The characteristics of MBD participants with alcohol consumption and without alcohol consumption were compared by Hillbom in a study. It was observed that the non-alcoholic participants with MBD were usually female and younger. It was always stated that the non-drinkers had malnutrition or frequent vomiting. The nutritional status of the MBD-affected alcoholics did not significantly differ from that of non-alcoholics. Only one alcoholic out of every 10 had a successful outcome, compared to more than half of the non-alcoholics who had fully recovered. However, the percentage of fatalities was roughly comparable between the two groups [7]. In a 2004 review by Heinrich et al., the patients were separated into two groups out of which group A had the worst cases. Their symptoms were more severe, and they also had severe impairment in consciousness with poor MRI results. The death rate of these patients was 21% and even the ones who survived had significant problems. Whereas group B had minimal deficits with no deaths and the patients had a favorable recovery. Only 20 (8%) of 250 patients in a sizable published series showed a satisfactory recovery [7]. Alcoholic MBD was linked to a worse result, and infectious complications were the major cause of mortality. A patient may continue to have symptoms for years, totally recover, go into a vegetative state, or pass away. The superior commissure fibers are thought to be relatively spared in partial lesions, which is associated with a better prognosis [20]. Cortical lesions (cortical involvement in acute MB disease), low apparent diffusion coefficient values of the cerebral cortex, involvement of the entire cerebral cortex, and severe disturbances of consciousness are just a few of the indicators of poor prognosis that have been suggested [35]. Patients who receive early diagnosis and treatment and who have circumscribed lesions have a better prognosis. Another important prognostic indicator is MRI which can show us a clear picture of clinical recovery and neuroradiological changes [1].

## Conclusions

MBD is a rare demyelinating disease that arises commonly as a complication of alcohol abuse for a long stretch of time. The lesions of this disease are characteristically present in the corpus callosum. The histological examination of the lesions shows necrosis, histocyte invasion, demyelination, and macrophages. The clinical stages of the disease can be seen clinically as acute, subacute, or chronic, with an array of symptoms and signs present in each stage. Initial symptoms can be as vague as apathy, headache, emotional and psychotic symptoms, and dizziness and the acute phase usually presents confusion, dysarthria, ataxia, limb hypertonicity, and delirium/coma. MRI is the current gold standard for the diagnosis of the disease. A distinct “sandwich sign" has been observed which is a valid confirmation of the disease. The lesions seen on T1W1 are hypointense, T2W1 are hyperintense, and FLAIR shows reduced diffusion in the central layer of the corpus callosum. It is tough to differentiate the disease from other alcohol use disorders but is of extreme importance for the treatment. The current treatment regimen for MBD is not concrete, but the general course includes thiamine therapy to replenish thiamine in the body. Steroids are also given to reduce inflammation and stabilize the blood-brain barrier. The outcome of the disease is variable, and generally, involvement of the cortex, low apparent diffusion coefficient values of the cerebral cortex and disturbances in consciousness are indicators of poor prognosis. It is of extreme importance to diagnose the disease in the initial stages and start immediate intervention while keeping track of lesions by various imaging methods. All the recent data that have been collected do not offer a conclusive treatment plan which can lead to the occurrence of preventable complications and deaths hence it is imperative to improve upon this in the future to better predict the outcome of the disease.
